# Anaphylactoid Reaction from IV Contrast Dye Causing Ischemic Colitis with Portal Venous Gas

**DOI:** 10.1155/2015/793951

**Published:** 2015-04-23

**Authors:** Adeleke Adesina, Adam Colombo, Rebecca Jeanmonod

**Affiliations:** St. Luke's University Hospital, 801 Ostrum Street, Bethlehem, PA 18015, USA

## Abstract

Portal venous gas is a radiographic finding with numerous causes. The most common etiologies include bowel ischemia or other intra-abdominal catastrophes. The finding of portal venous gas carries a high mortality rate. We report the first case of portal venous gas associated with anaphylactoid reaction to intravenous contrast dye in a middle-aged woman. This was likely secondary to anaphylactoid-induced ischemic colitis. This patient was managed conservatively and had a good outcome.

## 1. The Case

A 53-year-old female presented for an elective outpatient computed tomography (CT) scan of her abdomen and pelvis for longstanding inguinal pain. While receiving her injection of intravenous (IV) contrast, she developed sudden onset shortness of breath, severe constrictive chest and neck pain, and diffuse abdominal pain. She also had associated nausea and vomiting. While in the CT suite, she received a dose of intramuscular epinephrine and IV diphenhydramine. She was subsequently transferred to the emergency department (ED).

In the ED, the patient continued to complain of chest pain and abdominal pain. Her review of systems was otherwise unremarkable. The patient's past medical history was notable for asthma, hypertension, gastric ulcers, gastroesophageal reflux disease, and transient ischemic attack.

On physical examination, the patient was obese and anxious. Her heart was regularly tachycardic without murmurs or rubs. Her lungs were clear and equal with good air movement. Her abdomen was soft and nontender, with no guarding or rigidity. The remainder of her exam was unremarkable. The patient's laboratory values demonstrated a white blood cell count of 13.81 with neutrophil count of 10.08, sodium 134 mmol/L, potassium 2.9 mmol/L, chloride 97 mmol/L, and blood glucose 232 mg/dL. The remainder of her labs was normal. Her EKG showed no abnormalities and her chest X-ray showed mild cardiomegaly.

**Figure 1:**
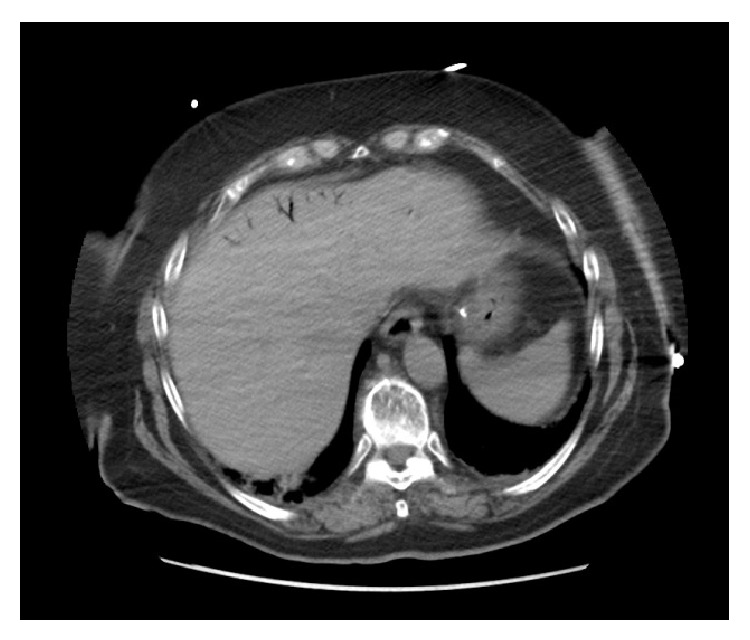
CT image demonstrating portal venous gas.

**Figure 2:**
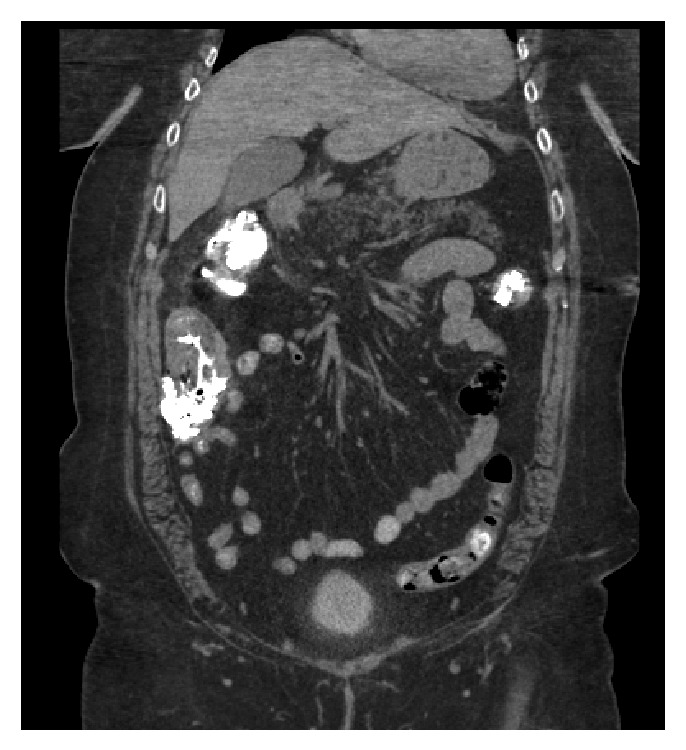
Focal thickening of ascending colon.

While in the ED, the patient was medicated for her pain and nausea with IV narcotics and ondansetron. She developed transient hypotension with a blood pressure of 84/62 and was fluid-resuscitated with 2 L normal saline. In spite of her treatment, the patient continued to complain of chest and abdominal pain. Repeat CT scan of her chest, abdomen, and pelvis was performed which demonstrated portal venous gas along with gastric wall edema and stranding fluid adjacent to the gastric antrum (Figure 1). There was thickening of the ascending colon above the ileocecal valve along with adjacent extraluminal gas which was thought to be within a small mesenteric vessel (Figure 2). These findings were new as compared to the CT completed three hours earlier.

Based on these CT findings, surgery was consulted. The patient continued with benign abdominal exam and stabilization of her vital signs, so conservative management was undertaken. On hospital day 2, repeat CT scan showed resolution of portal venous air as well as all inflammatory findings. On hospital day 5, the patient underwent colonoscopy which demonstrated mild ischemic colitis. The patient was ultimately discharged home in good condition.

## 2. Discussion

Wolfe and his coresearchers first described venous portal gas in a newborn diagnosed with necrotizing enterocolitis in 1955 [1]. More such cases were encountered thereafter [2]. Since then, gas in the portal vein has been considered an ominous sign and is mostly associated with serious underlying pathology. It has a high degree of mortality, once detected. However, this mortality is directly associated with the underlying pathology, as compared to the presence of the sign itself. There are many possible etiologies of portal venous gas, mostly affecting the gastrointestinal tract [3, 4]. Significant considerations include gastrointestinal (GI) ischemia, colitis, thrombosis, intestinal infection and perforation, GI carcinomas, ulcers, intestinal obstruction, and inflammatory bowel disease [4]. In our patient, portal venous gas was likely secondary to ischemic colitis.

Acute ischemic colitis may be secondary to venous or arterial occlusion or due to hypoperfusion from low-flow states (nonocclusive ischemic colitis), such as systemic hypotension, cardiac failure, and septic shock [5]. Ischemia causes edema and necrosis to the mucosal barrier of the bowel mucosa, leading to bowel dilatation as well as disruption of protective barriers. This allows gas to escape into bowel venous channels, which then accumulates in the venous system. Occlusive ischemia typically involves the descending colon [6]. This pattern is different than that seen in nonocclusive ischemic states, in which the right side of the colon is involved in the majority of cases [7, 8]. Our patient had ascending colonic thickening, supporting a diagnosis of nonocclusive ischemic colitis, which was confirmed on endoscopy.

Anaphylaxis (IgE mediated) and anaphylactoid (mast cell/basophil mediated) reactions are both immediate systemic reactions to foreign substances [9]. Skin, respiratory, cardiovascular, and gastrointestinal system involvements are all common. In spite of frequent gastrointestinal symptoms such as vomiting, diarrhea, and crampy abdominal pain, mucosal lesions and gastrointestinal ischemia are rarely detected [9]. There have been 2 cases of gastric mucosal lesions following anaphylaxis [9, 10] and 2 cases of anaphylaxis-mediated ischemic colitis and proctitis [8, 9]. Animal experimental studies suggest that mucosal anaphylaxis may cause gastrointestinal ulceration, and in fact the gut is the main organ of anaphylactic shock in rats [11–13]. However, in humans, there is no evidence that the gut mucosa is directly damaged by the immune response, but rather the mucosal findings are secondary to hypotension. These findings could conceivably be made worse by concomitant use of epinephrine, leading to vasoconstriction of the splanchnic bed.

Diagnosis of ischemic gastrointestinal disease is usually made by history and CT scanning, in association with other laboratory tests, such as lactate. Although surgery is usually undertaken in patients with a finding of portal venous gas, it is important to take into account the entire clinical picture. In patients with ischemic colitis associated with anaphylaxis/anaphylactoid reactions, aggressive resuscitation and careful monitoring may be the preferable option.

## 3. Conclusion

Ischemic colitis is a rare complication of anaphylactoid reactions and may present with the radiographic finding of portal venous gas. These patients warrant aggressive resuscitation and surgical consultation but may not require operative intervention.
